# Immigrants’ perspectives on healthy life and healthy lifestyle counseling: a focus group study

**DOI:** 10.1177/14034948221075021

**Published:** 2022-02-08

**Authors:** Maliheh Nekouei Marvi Langari, Jaana Lindström, Pilvikki Absetz, Tiina Laatikainen, Jussi Pihlajamäki, Tanja Tilles-Tirkkonen, Hannele Turunen

**Affiliations:** 1 Department of Nursing Science, University of Eastern Finland, Finland; 2 Finnish Institute for Health and Welfare, Finland; 3 Department of Public Health Solutions, Finnish Institute for Health and Welfare, Finland; 4 Department of Public Health and Clinical Nutrition, University of Eastern Finland, Finland; 5 Faculty of Social Sciences, Tampere University, Finland; 6 Collaborative Care Systems, Finland; 7 Institute of Public Health and Clinical Nutrition, University of Eastern Finland, Finland; 8 Joint municipal authority for North Karelia social and health services (Siun sote), Finland; 9 Public Health and Clinical Nutrition, Department of Medicine, University of Eastern Finland, Finland; 10 Endocrinology and Clinical Nutrition, Department of Medicine, Kuopio University Hospital, Finland; 11 Department of Clinical Nutrition, Institute of Public Health and Clinical Nutrition, University of Eastern Finland, Finland; 12 Kuopio University Hospital, Finland

**Keywords:** Health counseling, qualitative study, focus group, content analysis, immigration, health promotion, primary health care, health literacy, type 2 diabetes, chronic disease

## Abstract

**Background:**

Immigrants have a higher risk of developing chronic diseases than the general population, but there is a lack of knowledge about appropriate counseling models to promote their health. This study aimed to explore Asian and Russian immigrants’ perspectives in Finland on healthy lifestyle and healthy lifestyle counseling to improve the quality of the counseling in primary health care services to prevent type 2 diabetes and other chronic diseases.

**Methods:**

We conducted a qualitative study using semi-structured questions for eight focus groups. The participants were 46 adult immigrants (21 Asian and 25 Russian) living legally in Finland. Interviews were transcribed verbatim, coded, and analyzed using deductive content analysis.

**Results:**

We identified three themes regarding healthy lifestyle: (1) limited knowledge on different dimensions of healthy lifestyle, (2) understanding the impact of culture and community on healthy living, and (3) changing the lifestyle to live healthier after immigration. Moreover, we found three themes regarding healthy lifestyle counseling: (1) shortcomings in health care for providing healthy lifestyle counseling, such as lack of educational materials and miscommunication, (2) influential individual factors for using healthy lifestyle counseling, such as stress, and (3) positive outcomes of healthy lifestyle counseling.

**Conclusion:**

**Developing a culturally tailored healthy lifestyle counseling program for the enhancement of immigrants’ knowledge regarding healthy lifestyle with consideration of cultural and linguistic aspects is recommended for preventing chronic diseases among immigrants.**

## Background

Immigration is recognized as a global health challenge, and immigrants are at higher risk of developing chronic diseases [1,2]. Previous studies have confirmed the high incidence of diabetes at an earlier age among immigrants, in comparison to the native population [2–4] and the diabetes risk increases with years of residence in the host country [5]. Unhealthy eating and lack of physical activity are some of the factors that contribute to the incidence of type 2 diabetes (T2D) among immigrants [6].

Europe faced a historical immigration crisis in 2015, by receiving many asylum seekers, mainly from war-ridden countries in the Middle East. Likewise, the number of asylum seekers and immigrants has recently increased in Finland.

Finland is a country with a population of 5.5 million, including minority communities such as Sami, Roma, and Swedens, as well as immigrants. Immigrants embody nearly 8% of the total population of Finland, mainly from Estonia, Russia, Asia, and Africa respectively; with Russians and Asians being the biggest groups of non-European immigrants [7]. There is no health examination, screening, or medical recording of immigrants/refugees upon their arrival in Finland. Healthy lifestyle counseling is equally available for all residences in Finnish primary health care [8]. Counseling targets many health topics (physical, mental, etc.), age groups (child, adolescent, adult, etc.) and is performed via educational materials, individual visits or health promotion projects. However, it follows a relatively standard approach, designed for the major Finnish population, without regard for the cultural and linguistic diversity of immigrants. Hence, knowledge is scarce on immigrants’ perspectives of healthy lifestyles and how current healthy lifestyle counseling serves immigrants.

## Aims

As part of the Finnish project for T2D prevention among high-risk populations in Finland, Stop Diabetes [9], this study aims to explore the perspectives of Asian and Russian immigrants on healthy lifestyles and healthy lifestyle counseling in primary health care setting in Finland.

## Methods

This qualitative study with focus group interviews and semi-structured questions for Asian (A) and Russian (R) immigrants was conducted in 2017. Eight focus group interviews were conducted in six cities across Finland. Inclusion criteria were: age over 18 years and living in the country legally for at least 2 years. Study invitations were distributed in person or by mail to multicultural centers, Finnish language courses, at religious-cultural events. The research aims were explained to participants in plain language, and a connection was established with potential participants. Utilizing a purposive sampling method, we recruited immigrants across different age groups, genders, socio-economic status, and education levels to gather various perspectives on this subject. Focus group interviews were continued until data saturation was achieved. A total of 46 immigrants participated in the study, of which 21 participants were from different Asian countries, including Afghanistan, Iran, Syria, the Philippines, Bangladesh, Nepal, India, and Thailand. In addition, there were 25 Russian participants, mainly from Russia (*n* = 20) and other former Soviet Union countries (*n* = 5), such as Ukraine, Estonia, and Latvia, who recognized themselves more as Russian and spoke Russian as their native language. The time and location of interviews in public facilities was set with the participants in advance so everyone attended and no one bring a guest. Interviews were audio recorded and lasted for nearly 2 hours. The second moderator took field notes before and during the interviews and organized the interview sessions. Participants received a gift bag containing healthy foods worth 10 euros.

### Theoretical framework

To develop the interview questions and analyze the results, we used Bandura’s social cognitive theory, which explains how human behavior is shaped by a dynamic and reciprocal interaction between personal, behavioral, and environmental aspects [10]. Accordingly, interview questions were concerning the experience of healthy lifestyle counseling based on the individual, organization or living environment, and healthy behavior.

Following the guidelines of Kallio et al. [11], we initially developed the interview questions in English and then piloted them. Subsequently, they were translated into the Russian and Farsi languages. The interview language was Russian for four Russian groups, Farsi for two Asian groups (participants from Iran and Afghanistan), and English for two Asian groups (participants from other Asian countries). Each interview was conducted by two bilingual moderators, the first author and peer researcher, in either English, Russian, or Farsi.

The participants completed background questionnaires regarding gender, age, length of residency, education level, Finnish language skills, working status, frequency of using health care services, self-assessment of their own health, and the presence of a healthy lifestyle. Moreover, participants completed the Finnish Diabetes Risk Score (FINDRISC) [12] and the European Health Literacy Survey-Europe (HLS-EU) [13] as part of their background information. We used back-translation method for these questionnaires.

The FINDRISC test has eight questions with a score range of 0–26. The risk of developing T2D is low when the score is lower than 12, moderate if the score is 12–14, and high when the score is higher than 14. To assess health literacy (HL), we used the shortest version of the HLS-EU with 16 questions on a 5-point Likert scale in the three domains of health care, disease prevention, and health promotion. The total sum range was between 0 and 16, and we assigned “very easy” and “easy” a score of 1 and “difficult” or “very difficult” as 0 for each item. The HL total scores were classified into three levels: below 9 was inadequate, 9–12 was limited, and 13–16 was sufficient.

### Data analysis

The results of the demographic information, FINDRISC, and HLS-EU surveys were analyzed using SPSS Statistics version 25 (IBM Corporation) to determine frequencies and means. For analysis of the focus group interviews, we transcribed the verbatim audio recordings. Thereafter, two Farsi and four Russian interviews were translated into English by native and bilingual peer researchers. The first author (bilingual in English and Farsi) was a moderator in most of the interviews, and read the complete interview transcripts several times to become familiar with the entire data. Discussions were conducted with the Russian peer researcher to ensure full comprehension of the meaning and unclear expressions prior to coding and data analysis. With peer debriefing, we checked the entire research process and discussed the interview findings rigorously. Initial codes were generated by the first author and research group members held several meetings to classify the codes, resolve discrepancies, and perform the coding process multiple times to reach the final codebook, sub-categories, categories, and themes. Results were reported after full review, and mutual agreement between researchers was obtained. We analyzed the data using a deductive content analysis approach and the QSR NVivo software version 11 (QSR International).

The National Institute for Health and Welfare in Finland provided ethical clearance for the study (THL/949/6.02.01/2017). Participants gave oral informed consent after reading or hearing the study information before the interviews. We considered organizing gender-specific focus group interviews to respect the cultural and religious characteristics of participants; however, they expressed comfort in participating in a mixed gender focus group interview. As a result, we held only mixed-gender focus group interviews.

## Results

### Participant characteristics, FINDRISC, and HLS-EU scores

The majority of the participants (89%) were women, and 39% of participants were between 40 and 59 years of age. Nearly 40% had been living in Finland for 2–5 years, and more than half of the participants (52%) had undergraduate or postgraduate education, while 4% (*n* = 2) were illiterate (no educational background in their native language). Only 30% of participants believed that they live a healthy lifestyle. The total FINDRISC scores indicated that nearly one-fourth (24%) of participants were at moderate risk and 24% were at high risk of developing T2D in later life. Moreover, the HL score showed that two-thirds of participants had limited and inadequate HL (Table I).

**Table I.** Characteristics of the Asian (*n* = 21) and Russian (*n* = 25) immigrants in Finland, 2017.

Items	Asian % (*n*)	Russian % (*n*)	Total % (*N*)
**Gender**			
Male	19 (4)	4 (1)	11 (5)
Female	81 (17)	96 (24)	89 (41)
**Age (years)**			
24-39	57 (12)	12 (3)	33 (15)
40-59	43 (9)	36 (9)	39 (18)
60-78	0 (0)	52 (13)	28 (13)
**Years of living in Finland**			
2-5	60 (12)	28 (7)	42 (19)
6-10	20 (4)	32 (8)	27 (13)
⩾11	20 (4)	40 (10)	31 (14)
**Education**			
Illiterate	10 (2)	0 (0)	4 (2)
Primary education	19 (4)	4 (1)	11 (5)
Upper secondary /vocational school	19 (4)	44 (11)	33 (15)
Under/postgraduate	52 (11)	52 (13)	52 (24)
**Finnish language skills**			
Not at all	5 (1)	4 (1)	4 (2)
Poor	52 (11)	41 (10)	47 (21)
Good	43 (9)	50 (12)	47 (21)
Excellent	0 (0)	4 (1)	2 (1)
**Working status**			
Full time/part time job	48 (10)	20 (5)	33 (15)
Unemployed	24 (5)	24 (6)	24 (11)
Pension	10 (2)	52 (13)	33 (15)
Student	14 (3)	4 (1)	9 (4)
Family/sick leave	5 (1)	0 (0)	2 (1)
**Using healthcare service**			
< 1 time a year	16 (3)	12 (3)	14 (6)
1–3 times a year	53 (10)	56 (14)	55 (24)
4–6 times a year	16 (3)	16 (4)	16 (7)
⩾ 7 times a year	16 (3)	16 (4)	16 (7)
**Self-assessment of health**			
Very good	10 (2)	0 (0)	5 (2)
Good	50 (10)	29 (7)	39 (17)
Moderate	35 (7)	50 (12)	43 (19)
Poor	5 (1)	21 (5)	14 (6)
**Self-assessment of having a healthy lifestyle (nutrition, exercise, smoking and drinking)**			
Yes	40 (8)	21 (5)	30 (13)
Somewhat	55 (11)	67 (16)	61 (27)
Diabetes positive (Type 1 or 2)	19 (4)	20 (5)	20 (9)
**FINDRISC**			
Low risk (<12)	71 (12)	35 (7)	52 (19)
Moderate (12–14)	17 (3)	30 (6)	24 (9)
High (>14)	12 (2)	35 (7)	24 (9)
**Health Literacy**			
Inadequate (<9)	24 (5)	36 (9)	30 (14)
Limited (9–12)	43 (9)	32 (8)	37 (17)
Sufficient (13–16)	33 (7)	32 (8)	33 (15)

FINDRISC, T2D risk assessment to assess the risk for developing T2D within 10 years; T2D, type 2 diabetes.

### Healthy lifestyle

Three themes related to healthy lifestyle were identified in the analysis of the qualitative data (Table II).

**Table II.** Perspectives of the Asian (*n* = 21) and Russian (*n* = 25) immigrants on the healthy lifestyle.

Healthy lifestyle		
Sub-categories	Categories	Themes
Nutrition Exercise Sleep Alcohol and tobacco Age-related health challenges	Physical health	**Limited knowledge on different dimensions of healthy lifestyle**
Meditation Positivity Peaceful life	Mental health	
Stress Happiness	Emotional health	
Communication Friendship Social activities	Social health	
Sun Nature Availability of more unhealthy choices	Environmental health	
Food Income Social and health care	Different life quality at home and host country	**Understanding the impact of culture and community on healthy living**
Family-centered culture	Dual role of family in living healthy	
Women prioritizing family over themselves		
Unhealthy childhood habits		
Unhealthy food culture induced by war	Negative influential factors on food culture and health	
Low self-care		
Lower priority for health promotion	Misconceptions toward health	
Mental health stigmatization		
Importance of physical health		
Diet Physical activity	Positive change for living healthy	**Changing the lifestyle to live healthier after immigration**

#### Limited knowledge on different dimensions of healthy lifestyle

All participants possessed some information related to healthy or unhealthy living in physical, mental, emotional, social, and environmental dimensions; however, their knowledge was scattered and limited. No details or specific information were provided on any of the topics mentioned, and participants discussed general facts, sometimes without certainty or reliable references.

Many participants discussed the importance of healthy eating and taking a variety of foods including more vegetables, eating more frequent and smaller portions of food, participating in regular physical activity, sleeping enough, and avoiding tobacco, alcohol, or drugs. Moreover, participants detected unhealthy trends for different age groups in society, mentioning obesity and drug abuse as more prevalent health challenges among young people, and sedentary lifestyles and immobility among the older population.

“For me, diet and sport are in the first place for living healthy.” (R 2)

Many Asian participants indicated the importance of mental health in being healthy, and hence practiced meditation, positive thinking, and living peacefully.

“Healthy lifestyle for me is to think positive, always use this way of thinking for myself, so everything will be all right.” (A 5)

Participants described emotional health in terms of being happy and lower stress levels in their daily lives.

“Stress is a very influential factor in life of immigrants in the host country; I have witnessed stress among mothers, youngsters, etc. here.” (A 18)

In terms of social health, some explained how loneliness is a health threat in Finland, particularly during the winters; participating in social activities, making friends, and communicating more may help tackle this issue.

“Yes, to meet people especially in winter in Finland. They (Finns) do not talk too much. It is really important for me, not to be alone, to meet people, to talk, to communicate. . .It is very important.” (R 13)

With regard to the environment and health, some participants described how they suffer from the lack of sun; however, the peaceful and clean environment and air of Finland positively influenced their health.

“I have [a] garden, blueberries, lingonberries, these sort of things. What it’s called, quiet hunting, for mushrooms, berries, you know, also fishing, yes, I like it.” (R 3)

#### Understanding the impact of culture and community on healthy living

Many participants discussed the effect of cultural differences between the host country (Finland) and their native countries and its impact on their health. Some participants acknowledged the distinct and higher quality of living in Finland compared to their home country, in terms of the availability of various foods, higher income, better social and health services, and a cleaner and peaceful natural environment, as unhealthy eating habits had developed in their lives due to poverty, war, and poor access to healthy alternatives.

“When I was living in (my) home country, I did not have an opportunity to eat properly, so to speak, because there was not enough money, and time to learn how to eat properly. But here, for example, I got the opportunity to buy healthy products, and also, to learn more. . .” (R 6)

The dual role of family in healthy living was highlighted, as some participants discussed the positive role of family in shaping and practicing healthy habits, while others pointed out the difficulty of making healthy choices within their family when other members have different opinions. Many Russian women noted how a strong family centered culture pushed them to prioritize family desires, even unhealthy ones, over their own desires. In their culture, women work hard both outside and inside the home, resulting in sacrificing their own health and desires for the family’s unhealthy habits. Some participants described how unhealthy childhood habits shaped and affected their present health behaviors, such as being “chubby” as a child was valued as being healthy and pretty in their culture.

“. . .if you were chubby, then you were beautiful, [laughing], I was the prettiest girl. . .” (R 9)

We also found negative factors influencing food culture and health. Those who experienced a war in their native countries attributed their present unhealthy lifestyle to the unhealthy food culture induced by war. The unavailability of various foods, lack of vegetables, and inadequate information on healthy lifestyle created unhealthy habits that continued to prevail even now. Some participants mentioned that they had to survive, fulfilling their basic needs during wartime.

In some cultures, self-care was considered selfish and was not valued, leading some participants to ignore physical and mental self-care by prioritizing their children or family over themselves.

“You know, we never threw anything away, took the last piece. We are the children of war, that is why it is very important for us, you know. I would rather take it, than waste it. . .” (R 15)

Several participants asserted some misconceptions about health. Taking health promotion actions in the absence of any disease was an unfamiliar concept with low priority for some participants, and seeking mental health care was considered unacceptable in some cultures. Only a few participants believed that the concept of health is limited to the human body and the absence of any disease.

“In my culture, maybe it is somehow difficult to seek for a doctor for a mental disease. Yes, it is not easy to talk about something that is inside. Maybe it’s hard for someone to talk about it.” (A 8)

#### Changing lifestyle to live healthier after immigration

Participants reported mainly a positive change toward healthy living post immigration as their financial status improved with better accessibility to healthier foods and exercise. However, some participants continued to find it difficult to make healthy eating choices, highlighting that the abundance of attractive, unhealthy products and sweets in Finland unconsciously influences their choices and compels them toward unhealthy eating.

“Yes, I also started to eat more fruits and vegetables after I moved to Finland. . .” (R 1)

### Healthy lifestyle counseling

#### Shortcomings in health care for providing healthy lifestyle counseling

The majority of Asian and Russian participants affirmed that they did not experience any general healthy lifestyle counseling in the Finnish primary health care, with the treatment approach being the main focus of the Finnish health care system (Table III).

“Have not faced it, just because it does not exist.” (R 16)

**Table III.** Healthy lifestyle counseling at primary health care in Finland as perceived by Asian (*n* = 21) and Russian (*n* = 25) immigrants, 2017.

Healthy lifestyle counseling		
Sub-category	Category	Theme
Not experiencing general healthy lifestyle counseling	Organizational/environmental	**Shortcomings in the health care for providing healthy lifestyle counseling**
Specific health counseling only available to patients		
Lack of educational materials with different health contents and their non-translation to different languages		
Miscommunication		
Lack of a uniform counseling method in all units		
Accessibility of counseling services in different cities		
Prominent role of other health related sectors in providing healthy lifestyle counseling		
Low awareness of health-related facilities and services	Personal	**Influential individual factors for using healthy lifestyle counseling**
Strong demand for having healthy lifestyle counseling		
Positive attitude toward healthy changes		
Native community as a role model		
Stress		
Iatrophobia		
Making positive changes in life	Behavioral	**Positive outcome of healthy lifestyle counseling**

Some participants, however, received healthy lifestyle counseling from different healthcare providers (i.e., private health care). Several participants reported receiving specific health counseling related to their diagnosed chronic diseases, such as diabetes, hyperlipidemia, hypertension, and obesity.

A lack of health-related educational materials and tailored advertisements for services promoting healthy lifestyle was reported. Most of the current materials were considered outdated, limited to a few diseases, and not in their native language. Miscommunication with healthcare professionals often occurred while using health services in Finnish.

“I have not seen any educational materials that were provided, nothing. . .” (A 20)

Although a few participants related direct experiences with special health counseling services, no uniform counseling approach was reported in terms of the content, organization, and follow-ups. Participants believed that no real health counseling was provided to them, as counseling was primarily an informal advice given during appointments rather than actual discussions with healthcare professionals at separate times covering different health promotion subjects. A few participants mentioned that healthy lifestyle counseling services varied between cities, with the quality and vicinity of these counseling services to home being important issues determining their utilization, particularly in big cities. The prominent role of other sectors in providing healthy lifestyle counseling was highlighted by some participants who had used private sector counseling services, Finnish language courses, and non-governmental patient organizations, such as the Finnish Diabetes Association.

#### Influential individual factors for using healthy lifestyle counseling

Many participants acknowledged their low awareness of available health counseling services, specifically in primary health care, while they expressed the strong demand in their community to receive healthy lifestyle counseling. Increased personal motivation was mentioned as a determining factor for benefiting from any health services, including healthy lifestyle counseling. In addition, participants exhibited a positive attitude toward making healthier choices to live longer and healthier.

“No one provided us with information. You find something (randomly), somewhere, read it. . .” (R 10)

The role of the host society was highlighted as a few participants praised the healthy lifestyles of Finnish people as a good model to follow.

“Finnish diet is healthy, look at 70 years old here how healthy they are!” (A 23)

However, deterrent factors such as stress and iatrophobia were identified regarding using healthcare services.

#### Positive outcomes of healthy lifestyle counseling

Most participants who had experienced any type of health counseling, either specific or general, found it effective to make healthy lifestyle changes later. They claimed they reduced sugar consumption, eat more vegetables, and exercise daily according to instructions given by healthcare professionals.

“Well, I’ve been visiting [a] dietician for a few months. . .I was losing weight with them. . .” (R 19)

“I changed my diet, which is good for me, and exercise more often. Yes, they told me so.” (A 17)

## Discussion

Overall, we found three main themes regarding healthy lifestyles among immigrants in Finland: (1) participants had limited and scattered knowledge on different dimensions of healthy lifestyle, (2) culture played an important role in shaping healthy habits and lifestyle, and (3) participants experienced lifestyle changes after immigration, mostly toward a healthier lifestyle. Three main themes also emerged regarding healthy lifestyle counseling: (1) shortcomings in the health care system, (2) individual factors contributing to the use of health counseling services, and (3) positive outcomes of healthy lifestyle counseling.

One of the main findings was that participants exhibiting limited and scattered knowledge on different dimensions of healthy lifestyle. Despite additional background investigations confirming that nearly half of the participants had undergraduate and postgraduate education, only 32% showed sufficient HL. HL is defined as “people having the appropriate skills, knowledge, understanding, and confidence to access, understand, evaluate, use, and navigate health and social care information and services.” [14]. Previous studies in non-immigrant populations have shown that low HL is associated with low education and poor health outcomes [15]. A Norwegian study reported low HL among immigrant populations in general, also associated with their low awareness of health care services in the host country, poor health status, and difficulty in making healthy lifestyle choices [16]. HL in the three domains of health care, disease prevention, and health promotion [17] is a prerequisite for any health-promoting service for immigrants to improve health outcomes [18,19] and decrease inequalities [14]. Regardless of an immigrant’s education, this gap needs to be addressed by advocating for health information presented in various languages for immigrants [14,16].

The present study emphasizes understanding the impact of immigrants’ cultural background and family health habits in shaping their current lifestyles with regard to their health. Despite most participants having resided in Finland for more than 6 years, nearly 50% of them had little to no Finnish language skills, seriously restricting their participation in health promotion programs, ability to receive adequate information, and use of different health services. Immigrants tend to be less physically active than natives [20] and their lifestyle is associated with developing diabetes later in life [21]. Hence, more emphasis needs to be placed on familiarizing the immigrant population with the healthy lifestyle practices in the host country (i.e., Nordic diet and routine physical activity) and western culture, where citizens have become empowered and autonomous in making healthier decisions for themselves and their family members. Strong connections with family and culture can be useful if the family adopts and supports healthy habits. Integration and acculturation are predictors of higher health literacy [16] and better health outcomes among immigrants [20,22]. Many of our participants had no experience of primary health care centers’ general lifestyle counseling in various cities. Hence, our findings, rather than presenting the participants’ experience of healthy lifestyle counseling, provide a general perspective on healthy lifestyle counseling for immigrants, including their expectations and reinforcement factors.

Stable economic conditions, better access to sport facilities and healthier food products, and the peaceful environment of Finland have made it conducive for participants to live a healthier life after immigrating from their native countries. Participants who received special lifestyle counseling related to their chronic disease reported a positive change in their lifestyle, reflecting their improved motivation, self-care, and choosing a healthier lifestyle.

In Finland, municipalities are responsible for the health and well-being of their citizens and assuring equality and accessibility of all health care services in the region for all population groups [8]. Despite the best efforts of public health care centers in different target municipalities, health promotion services provided for the general population did not reach the minority groups of immigrants efficiently, and were largely restricted to lifestyle counseling for people with chronic diseases.

According to the Ottawa Charter for Health Promotion, more intersectoral actions should be undertaken by building health policies supporting immigrants and strengthening the cultural competence of healthcare to address immigrant clients’ needs [23]. Community empowerment is an effective way to improve the health behaviors of minority groups such as immigrants, and to reduce health inequalities. The participatory nature of actions, power sharing, cultural adaptation, bicultural community healthcare workers, and collaboration of different partners are key factors in achieving better health outcomes [24]. Moreover, a culturally adapted healthy lifestyle intervention has been found to be an effective tool for reducing the incidence of T2D among immigrants [6,25].

Worldwide, immigrants visit healthcare services less frequently than native populations, which leads to inequality in service usage in society [26,27]. Immigrants tend to give low priority to health promotion, [26] and their cultural and health beliefs may become obstacles to utilizing available preventive services [26,28]. Although participants highlighted individual factors such as positive motivation and feeling the need for healthy lifestyle counseling in reinforcing the pursuit of preventive health services, their awareness of services available in society was low. Thus, effective methods in health promotion, such as social marketing, can be used to raise awareness of risky behaviors, increase motivation, and to support lifestyle change.

### Strength and limitations

This large-scale qualitative study with focus group interviews is among the few studies in Finland reflecting the voice of the biggest group of immigrants (Asian and Russian) on health promoting services and healthy lifestyle counseling to prevent T2D and other chronic diseases. To obtain broader perspectives on this less studied subject, we created heterogeneous focus groups (age, education, gender, etc.) that shared the same culture, language, and immigration experience.

The first limitation of this study is related to the purposive sampling method, which restricts the transferability of our findings. Second, although we recruited participants from different backgrounds, ages, education levels, and residential areas to cover different perspectives, these findings might not cover the totality of the immigrant population. Third, we used three interview languages because of the difficulty in interviewing participants in various native languages. As a result, translation may have affected the accuracy of the meaning of the participant responses. However, we tackled this challenge by including participants who were able to express themselves openly in English language, using peer checking and analyzing the data via multiple discussions between researchers, interviewers, and translators. Finally, since the majority of our study participants were women, our findings may largely represent the perspectives of women. More studies with in-depth individual interviews from different immigrant groups and genders are recommended to increase the knowledge regarding the healthy lifestyle counseling service in the host countries.

## Conclusions

Sustainability of healthcare, prevention of chronic diseases, and elimination of health inequalities are the primary targets of European health care [29]. Diabetes is considered an emergent condition of this century, and nearly half of the population living with diabetes has not yet been diagnosed [30]. Healthy lifestyle counseling services at primary health care facilities require cultural adaptation to reach immigrant clients. Diversity in the background, culture, social class, work, and education level of immigrants puts them at risk of inequality related to service usage and lowers health outcomes. Addressing health literacy, an overlooked factor in the health profile of immigrants, as well as implementing culturally tailored health-related information and health promotion programs and campaigns to prevent chronic diseases, including T2D, are recommended. Moreover, primary healthcare professionals dealing with immigrants need to be culturally sensitive and competent to provide client-centered services. The study findings can be used as a bottom-up approach to develop effective, culturally tailored, healthy lifestyle counseling for target immigrants.

## Supplemental Material

sj-docx-1-sjp-10.1177_14034948221075021 – Supplemental material for Immigrants’ perspectives on healthy life and healthy lifestyle counseling: a focus group study Click here for additional data file. Supplemental material, sj-docx-1-sjp-10.1177_14034948221075021 for Immigrants’ perspectives on healthy life and healthy lifestyle counseling: a focus group study by Maliheh Nekouei Marvi Langari, Jaana Lindström, Pilvikki Absetz, Tiina Laatikainen, Jussi Pihlajamäki, Tanja Tilles-Tirkkonen and Hannele Turunen in Scandinavian Journal of Public Health
