# Supplementary material for: Immigrants’ perspectives on healthy life and healthy lifestyle counseling: a focus group study
Source: Scand J Public Health. 2022 Feb 8;51(3):371–80. doi: 10.1177/14034948221075021 (PMC10251456; doi:10.1177/14034948221075021)
Supplement: sj-docx-1-sjp-10.1177_14034948221075021 – Supplemental material for Immigrants’ perspectives on healthy life and healthy lifestyle counseling: a focus group study [file sj-docx-1-sjp-10.1177_14034948221075021.docx]

**Interview questions:**

- What do you think is a healthy lifestyle?

-What does a healthy lifestyle include?

- Have you received a healthy lifestyle counseling in Finland? If yes, explain how was your experience of the received lifestyle counselling in Finland? From where/whom/what kind of information did you receive?

-How effective/clear/satisfactory did you find the health counseling to make any change in your lifestyle to become healthier)?

-In general, what are the influential factors for having healthy lifestyle?

-What is the role of the individual (i.e., culture, language, motivation, etc.) regarding receiving the healthy lifestyle counseling?

-What is the role of healthcare and other organizations in providing healthy lifestyle counseling?

-What are the environmental factors (i.e., housing area, community, facilities, climate etc.) influencing receiving the healthy lifestyle counseling?
